# Accelerating the implementation of digital pathology in India

**DOI:** 10.1016/j.jpi.2026.100664

**Published:** 2026-04-30

**Authors:** Sangeeta Desai, Jayesh Deshmukh, Anurag Mehta, Kunal Sharma, M.N. Vidya, Kirti Chadha, R. Veena, Sandeep Sewlikar, Ana Richelia Jara-Lazaro

**Affiliations:** a Tata Memorial Centre, Dr. E Borges Road, Parel, Mumbai 400012, India; b Roche Diagnostics India and Neighboring Markets, 501B, Silver Utopia, Cardinal Gracias Road, Chakala, Andheri East, Mumbai 400069, India; c Rajiv Gandhi Cancer Institute and Research Center, New Delhi 110085, India; d Global Reference Laboratory, Agilus Diagnostics, Plot No 1, Prime Square Building, Gaiwadi Industrial Estate, Next to Patel Petrol Pump, Opposite Mahesh Nagar, S.V. Road, Goregaon West, Mumbai 400062, India; e Manipal Hospitals, 98, HAL Old Airport Rd, Kodihalli, Bengaluru 56001, India; f Metropolis Healthcare Ltd., 4th Floor, East Wing, Plot-254 B, Nirlon House, Dr. Annie Besant Road, Worli, Mumbai 400030, India; g HCG Cancer Hospital, No 8, HCG Towers, P. Kalinga Rao Road, Sampangi Ram Nagar, Bangalore 560020, India; h Roche Diagnostics India Pvt Ltd., 501B, Silver Utopia, Cardinal Gracias Road, Chakala, Andheri East, Mumbai 400069, India; i Roche Diagnostics Asia-Pacific Pte Ltd., 8 Kallang Avenue, #10-01/09 Aperia, Tower 1, 339509, Singapore

**Keywords:** Digital pathology, Whole-slide imaging, Artificial intelligence, Telepathology, Implementation barriers, Healthcare in India, AI-assisted diagnostics

## Abstract

Digital pathology (DP), which utilizes whole-slide imaging (WSI), adds value to traditional pathology by enabling high-resolution imaging, remote diagnostics, and artificial intelligence (AI)-assisted analysis. Despite its potential, DP adoption in resource-constrained settings, such as India, remains limited. A significant gap exists in understanding the diverse applications of DP, adoption barriers, and cost-effective implementation strategies specific to India's healthcare landscape. Hence, this narrative review explores opportunities for DP application in India, including primary diagnosis, second opinion, telepathology, education, frozen section analysis, AI-assisted diagnostics, data management, and quality assurance. It also examines the hurdles toward widespread adoption, including infrastructure and information technology limitations, high costs, and data security concerns. Limited awareness and apprehensions about job displacement further prevent DP adoption. Addressing these challenges requires a strategic approach, including innovative revenue-generating strategies, targeted training programs, robust data security, and development of high-quality datasets. Further, collaboration among pathologists, healthcare providers, policymakers, and technology developers is essential. With structured implementation and targeted investments, DP can potentially revolutionize pathology services in India, enhancing diagnostic accuracy, reducing turnaround times, and expanding access to specialized care, particularly in underserved regions.

## Introduction

Digital pathology (DP) refers to the acquisition, management, and interpretation of high-resolution digital images of histopathological glass slides using whole-slide imaging (WSI) scanners, integrated with an image management software and, increasingly, artificial intelligence (AI) algorithms.^1^, ^2^ Unlike conventional light microscopy, DP enables remote access to digital slides, facilitates AI-assisted image analysis, supports long-term archival, and eliminates the need for physical slide transportation, capabilities of particular relevance in geographically dispersed, resource-constrained healthcare systems.^1^, ^3^, ^4^ By reducing inter- and intra-observer variability and enabling pathologists in remote areas to access specialist opinion, DP has the potential to address longstanding equity gaps in diagnostic services.^3^, ^5^, ^6^, ^7^

Quantitative evidence supports these benefits: optimized DP workflows have been associated with reductions in turnaround time (TAT), with some institutions reporting sign-out times comparable to or faster than conventional microscopy.^6^, ^8^ Diagnostic concordance studies have consistently demonstrated that WSI is non-inferior to glass slide evaluation for primary diagnosis across multiple surgical pathology subspecialties.^9^

India's healthcare system is characterized by profound structural heterogeneity. It operates across a three-tier hierarchy, primary health centers, district or secondary hospitals, and tertiary or teaching institutions, with a coexisting and dominant private sector that delivers an estimated 70%–80% of outpatient care.^10^, ^11^ Geographically, this translates into a stark urban–rural divide: specialist pathologists and advanced diagnostic infrastructure are predominantly concentrated in metropolitan Tier 1 cities, whereas Tier 2 and Tier 3 cities, as well as rural districts, are critically underserved. Despite having one of the largest absolute numbers of pathologists globally, India faces a significant shortage of qualified pathologists relative to its population of over 1.4 billion, with the deficit most acute in rural and semi-urban areas, where many labs operate without a certified pathologist on staff.^12^, ^13^ This maldistribution is compounded by the high burden of both communicable and non-communicable diseases, a rapidly expanding diagnostics market, and significant variability in lab quality standards across institutions.^12^, ^13^, ^14^, ^15^, ^16^ It is within this complex landscape that DP must be implemented, not as a uniform, top-down technology deployment, but as a context-sensitive solution tailored to the specific needs, resources, and constraints of each tier of the healthcare system.

Whereas the concept of telepathology emerged in the 1980s, it gained popularity in the 1990s with the advent of automated WSI and supporting software.^17^, ^18^, ^19^ In India, the use of DP first started in 2000, when a static telepathology service was established between two hospitals in Barshi and Mumbai to bridge the gap between a medically underserved, remote rural area and an advanced center with technical expertise.^20^ The Indian diagnostics market was anticipated to reach USD 9 billion by 2025, growing at a compound annual growth rate of ∼10%.^21^ This growth, coupled with the increasing prevalence of non-communicable diseases and a shortage of specialized pathologists, presents a significant opportunity for the widespread adoption of DP in India, especially in rural and underprivileged regions.^12^, ^13^, ^14^, ^15^, ^16^

A critical knowledge gap persists in delineating the full spectrum of DP applications, as well as in systematically characterizing the barriers to its adoption and the implementation strategies required to address the unique structural, economic, and operational constraints of the Indian healthcare ecosystem. In particular, evidence remains limited regarding context-specific insights from resource-constrained settings and the feasibility of cost-effective deployment models. To address these gaps, the present narrative review provides a comprehensive and integrative assessment of: (i) the evolution of DP and its convergence with AI; (ii) the impact of digitization on diagnostic workflows, efficiency, and quality in pathology; (iii) key pre- and post-adoption barriers spanning technological, regulatory, infrastructural, and workforce domains; and (iv) potential solution pathways tailored to the Indian context. Distinct from prior reviews that predominantly focus on technological advancements or global adoption trends, this manuscript offers a novel, India-centric synthesis of implementation evidence, explicitly incorporating cost and infrastructure considerations, and advances a pragmatic, phased adoption framework designed to enable scalable integration across heterogeneous healthcare tiers characteristic of low- and middle-income country settings.

### Methodology

This narrative review was conducted using a structured and transparent literature search approach to enhance reproducibility. Electronic databases, including PubMed/MEDLINE, Scopus, and Google Scholar, were systematically searched for studies published between January 2000 and December 2025. The search strategy combined controlled vocabulary and free-text terms using Boolean operators. A representative search string included: (“digital pathology” OR “whole slide imaging” OR “WSI”) AND (“India” OR “low- and middle-income countries” OR “LMIC”) AND (“implementation” OR “barriers” OR “telepathology” OR “artificial intelligence” OR “machine learning”). Titles and abstracts were independently screened for relevance, followed by full-text assessment of eligible articles. Studies were included if they addressed applications, validation, implementation challenges, or adoption of DP, with prioritization given to Indian and low- and middle-income countries (LMIC) contexts. Exclusion criteria comprised non-English publications, conference abstracts without full datasets, and studies lacking relevance to clinical or implementation aspects. Although formal meta-analysis was not feasible due to heterogeneity, an estimated body of over 120 relevant articles was screened, of which approximately 65 were included for qualitative synthesis. A narrative synthesis approach was adopted, focusing on recurring themes in infrastructure, workflow integration, cost, and clinical validation. Potential selection bias was mitigated by cross-referencing citations and prioritizing multi-institutional and high-quality studies where available.

## Applications and opportunities for digital pathology in India

**Fig. 1:**
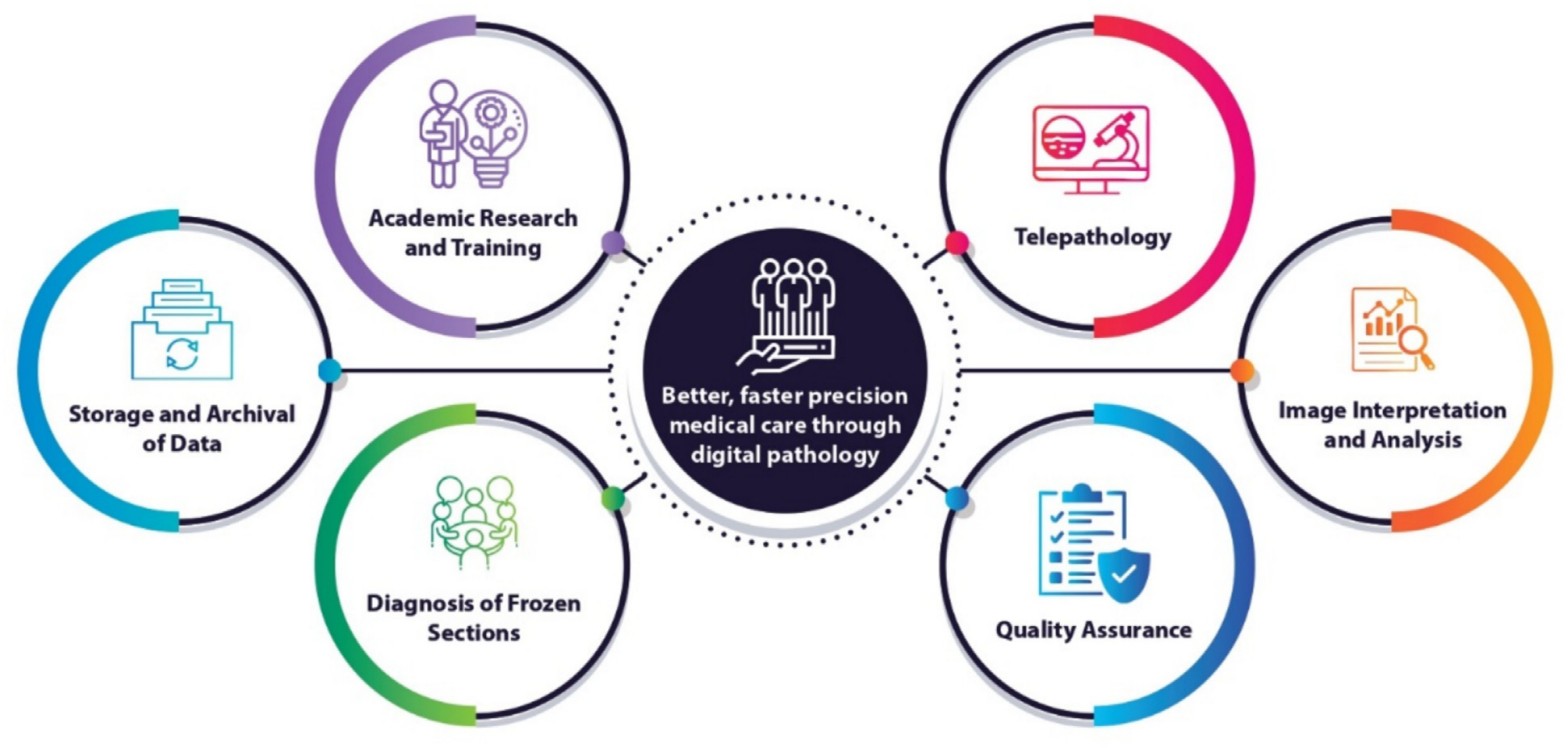
Applications of digital pathology in India.

The applications of DP in routine diagnostic practice span several clinical domains. These include primary histopathological diagnosis using WSI as a direct replacement for glass slide microscopy, intra-operative consultation through real-time telepathology for frozen section (FS) analysis, remote second-opinion consultations between institutions, including rural referral centers and tertiary academic hospitals, and subspecialty referral networks enabling expert review of complex or rare cases across geographic boundaries. Each of these applications holds promise in the Indian context, where specialist pathologists are concentrated in urban centers and large academic institutions, leaving peripheral and rural facilities underserved. The applications of DP are summarized in Fig. 1, and real-world use cases are listed in Table 1.

**Table 1:** Use cases of digital pathology in infrastructure, regulation, and tier feasibility in India.

Use case	Required infrastructure	Regulatory considerations	Tier 1 feasibility (metro/teaching hospitals)	Tier 2/3 feasibility (district/rural centers)
Education & training	• WSI scanner (basic capacity)^22^, ^23^ • Image management and annotation software^22^, ^23^ • Stable broadband (∼5–10 Mbps) • Digital microscope for live video streaming^22^, ^23^	• Institutional data governance for de-identified teaching datasets^22^ • Patient consent for use of images in education • NMC/NBE guidelines on digital teaching methods	High Teaching institutions with established IT; preferred by 94.3% of students in Indian studies; post-test scores improved significantly with digital method (*p* = 0.007)^24^, ^25^	Moderate Internet connectivity is the primary constraint; cloud-hosted WSI libraries reduce local infrastructure burden; CPCs via DP feasible remotely^27^, ^28^
Telepathology & remote reporting	• WSI scanner + digital workstation^1^, ^32^ • Reliable ≥10 Mbps connectivity for sign-out; higher for frozen section^29^ • Secure image transmission platform^32^ • DP–LIMS integration^64^ • MDT collaboration infrastructure^35^	• Telemedicine Practice Guidelines 2020 (NMC/NITI Aayog)^91^ • DPDP Act 2023: patient data privacy in transmission • NABL: concordance validation required before routine use	High Validated at Tata Memorial Centre (567 cases, 1.2% minor discordance, no major discordance); multiple Indian tertiary centers adopted during COVID-19^23^, ^29^, ^30^, ^31^	Moderate Network instability is critical barrier; static telepathology feasible at lower bandwidth; hub-and-spoke model most viable; first India service established 2000 (Barshi–Mumbai)^20^, ^36^
Primary WSI diagnosis	• High-capacity WSI scanner (≥100 slides; USD 100 K–400 K)^70^, ^71^ • Medical-grade display monitors (USD 5.5 K–39.5 K)^70^, ^71^ • Robust server and storage infrastructure^62^, ^63^ • LIMS integration and dedicated IT support staff^64^, ^65^	• NABL: mandatory analytical validation (concordance study, SOPs) • Medical Devices Rules 2017 if AI-assisted^92^ • CDSCO oversight for AI-embedded diagnostic workflows^92^	High WSI non-inferior to glass slide across surgical pathology; Indian studies: κ = 0.9 for prostate diagnosis; 66.22% concordance for tubular atrophy grading with eyeballing over-grading^9^, ^37^, ^38^	Low–moderate High capital cost of scanners and servers prohibitive for small labs; most feasible via centralized hub model; performance strongly dependent on institutional readiness^29^, ^37^, ^38^, ^61^, ^73^
Frozen section diagnosis	• Portable or high-speed WSI scanner^57^ • High-bandwidth connectivity for real-time streaming^55^, ^56^ • Coordinated team: histotechnologist, IT, surgeon, remote pathologist^54^, ^55^, ^56^	• Telemedicine Guidelines 2020 (remote intraoperative consultation)^91^ • Institutional validation protocol and SOPs mandatory^54^ • CAP-equivalent intraoperative TAT benchmarks to be met^54^, ^55^, ^56^	High Indian validation: 97.6% accuracy, κ = 0.993 inter-observer, κ = 0.987 intra-observer across 4 pathologists using portable scanner; TAT reduced from >20 to ∼15 min^54^, ^55^, ^56^, ^57^	Low High bandwidth and real-time reliability essential; skilled histotechnologists scarce; addresses greatest unmet need (absence of on-site pathologist) if connectivity solved^54^, ^57^
AI-assisted diagnosis & triage	• WSI infrastructure (as above) • GPU-enabled servers (USD 20 K–40 K)^70^ • AI/ML software platform (USD 20 K+ base)^70^ • Pathology informatics expertise^88^ • High-quality annotated training datasets^94^, ^96^	• Medical Devices Rules 2017 (amended 2020): AI as SaMD^92^ • CDSCO: no India-specific SaMD pathway yet defined^92^ • No equivalent to FDA clearance or CE marking currently available^41^ • Independent AI ethics committee oversight required^93^ • Informed patient consent for AI-assisted diagnostics^93^	Moderate Validated at Indian centers for HER2 dual ISH (non-inferior to manual scoring); AI Gleason scoring superior to pathologist-derived GS for risk stratification; regulatory pathway nascent^41^, ^43^, ^44^, ^45^, ^92^	Low GPU infrastructure cost-prohibitive; AI triage validated for cervical cytology and prostate biopsies: highest impact potential if cloud-deployed; AI models require India-specific datasets^47^, ^48^, ^94^, ^96^
Data storage & archival	• Tiered storage: SSD (active) + tape/cloud (archival); 15–200 TB/year for 50 cases/day^1^, ^35^, ^63^, ^68^ • DICOM-compatible PACS or linked repository^69^ • JPEG2000 compression pipeline^63^, ^68^, ^69^ • Retention policy management^1^, ^22^	• DPDP Act 2023: data localization, consent, and accountability • ABDM: federated health data exchange standards^79^, ^80^ • DICOM and HL7/FHIR standards for interoperability^66^, ^69^ • Institutional retention and deletion SOPs required^1^, ^22^	High Large centers have existing server infrastructure; Cancer Digital Slide Archive and TCGA models available; CHAVI biobank emerging in India^49^, ^50^, ^97^	Moderate Cloud solutions reduce on-premise burden; JPEG2000 enables remote archival at reduced bandwidth; DPDP compliance infrastructure needed; India Pathology Dataset nascent^68^, ^69^, ^96^
Quality assurance	• LIMS with end-to-end traceability and real-time monitoring^51^ • Point-of-use QA system for image quality assessment^52^, ^53^ • Standardized SOPs and documentation systems^52^, ^53^ • External QA program participation infrastructure	• NABL accreditation: QA framework mandatory for certified labs • No India-specific digital pathology QA standard yet (CAP/RCPath equivalent pending) • Professional bodies (IAPB) to lead national QA guideline development	High NABL-certified labs have baseline QA infrastructure; US institutions have pioneered DP-integrated QMS with gap analyses and action plans; DP extends and automates existing frameworks^51^, ^52^, ^53^	Moderate Many labs lack NABL certification; QA adoption contingent on DP deployment; standardized national programs essential to address variability in diagnostic quality across institutions^51^

Feasibility key: High, deployable with current institutional readiness; Moderate, feasible with targeted investment or hub models; Low, significant infrastructure, cost, or regulatory gaps remain.

ABDM: Ayushman Bharat Digital Mission; AI: artificial intelligence; CAP: College Of American Pathologists; CDSCO: Central Drugs Standard Control Organization; CHAVI: cancer imaging archive; CPCs: clinicopathological conferences; DP: digital pathology; DPDP Act: Digital Personal Data Protection Act; FDA: Food And Drug Administration; FHIR: fast healthcare interoperability resources; GPU: graphics processing unit; HER2: human epidermal growth factor receptor 2; HL7: health level seven; ISH: in situ hybridization; IT: information technology; LIMS: laboratory information management system; MDT: multidisciplinary team; NABL: National Accreditation Board For Testing And Calibration Laboratories; NBE: National Board Of Examinations; NMC: National Medical Council; PACS: picture archiving and communication system; SaMD: software as a medical device; SOPs: standard operating procedures; TAT: turnaround time; TCGA: the cancer genome atlas; WSI: whole-slide imaging.

### Education, training, and academic research

Teaching institutions can utilize the improved visualization offered by DP as a tool for education and knowledge dissemination between healthcare providers and students. WSI images are interactive and easy to share without the risk of fading and breakage. They can be annotated for clarity, can be used in presentations, and require minimal preparation time for conferences. Digital images can also be used to generate teaching sets for a wide range of cases, especially rare ones, providing hands-on experience with uncommon conditions that would otherwise be inaccessible in traditional settings. They can also be shared for self-paced or remote learning, enriching the educational experience.^22^, ^23^ WSI is increasingly being used in examinations and proficiency testing. Images of entire slides are provided to students to answer clinical questions (“on-the-spot”) at tumor boards. Further, digital microscopes facilitate live video streaming of slide reviews, offering valuable insights into histopathological evaluation methods for training.^22^, ^23^

Chaudhari et al. prospectively compared the effectiveness of digital and conventional teaching methods in surgical pathology for second-year medical students. Of them, 50.7% preferred the digital method for studying gross pathology, whereas 56.92% favored it for microscopic examination. Post-test scores significantly improved with the digital method (mean increase of 2.75, *p* = 0.007).^24^ Similarly, Nautiyal et al. compared the effectiveness of teaching surgical pathology to 91 final-year medical students using formalin-preserved specimens and digital methods. Notably, 94.3% of students preferred the digital method, and 90.8% reported improved understanding of the subject.^25^ Hande et al. compared the effectiveness of virtual and conventional microscopy in teaching dental histology. Students who used a combination of virtual and conventional microscopy demonstrated the highest knowledge gains and satisfaction compared to those using either method alone.^26^

Additionally, clinicopathologic conferences (CPCs) provide an invaluable opportunity for students and residents to engage in the logical analysis of differential diagnoses and patient management.^27^, ^28^ The use of DP in CPCs in India could foster interactive discussions and critical thinking, offering medical students an excellent opportunity to sharpen their diagnostic skills.

These findings collectively suggest that integrating digital tools with traditional methods could enhance students' understanding and engagement in pathology and histology education. The digital approach not only improves subject comprehension but also complements conventional teaching techniques, particularly in fostering clinical correlation and practical learning across diverse healthcare settings in India, from urban to rural areas.

### Telepathology: Remote reporting and expert opinion

From infancy, telepathology has become an adolescent and gained popularity in India during the COVID-19 pandemic due to increased accessibility to expert pathologists.^29^, ^30^, ^31^ Telepathology facilitates expert consultations, second opinions, and remote diagnosis, making it invaluable for public healthcare systems, especially in underserved regions.^3^ Given India's growing healthcare needs and shortage of pathologists, telepathology offers a promising solution.^12^, ^13^, ^14^, ^15^, ^16^ Our current understanding suggests that telepathology would present a significant opportunity for advancement in diagnostic services.

A key advantage of telepathology is its ability to overcome geographical barriers between expert and reporting pathologists.^1^ Traditionally, complex cases requiring expert opinions involve the physical transportation of glass slides or paraffin blocks, with accompanying risks, often delaying diagnosis and treatment. Telepathology eliminates this bottleneck by leveraging digital imaging workstations to capture high-resolution images and transferring them via telecommunication networks. The images are viewed remotely on screens, enabling patients to receive timely and efficient consultations.^32^

Multidisciplinary management is crucial for effective patient care, particularly in complex conditions like cancer. Most developing countries do not have specific guidelines for multidisciplinary teams (MDTs) or centers with formal MDTs. Currently, the approach of MDT meetings is limited to selected regional centers in India.^33^, ^34^ Telepathology could facilitate MDT meetings and help extend these services nationwide through the sharing of annotated high-resolution WSI images with diverse healthcare professionals (e.g., oncologists, pathologists, and radiologists) for clinical decision-making.^35^

Real-world examples from India and comparable LMIC settings illustrate the practical impact of telepathology in geographically dispersed contexts. The first telepathology service in India, established in 2000 between the Tata Memorial Centre in Mumbai and the Nargis Dutt Memorial Charitable Hospital in Barshi, Solapur, a medically underserved rural area, demonstrated that static telepathology could bridge a critical diagnostic gap between peripheral and specialist centers.^20^ More recently, during the COVID-19 pandemic, multiple Indian tertiary oncology centers validated remote reporting workflows using WSI, demonstrating that high diagnostic concordance could be maintained outside the traditional lab setting.^29^, ^30^, ^31^ In sub-Saharan Africa, telepathology programs have similarly demonstrated feasibility in settings with limited pathology infrastructure, underscoring the generalizability of this model to India's Tier 2 and Tier 3 cities and rural districts.^36^ These applications are not merely conceptual; their real-world implementation across Indian healthcare institutions is discussed in "Current Scenario and Impact of Digital Pathology in India" , where multiple centers have validated these workflows in clinical practice.

### Image interpretation and analysis

#### Traditional versus digital images

WSI has shown non-inferiority to traditional glass slide evaluation for primary diagnosis across surgical pathology specimens.^9^ Moreover, optimized DP workflows reduce manual errors, improve visualization, and ensure reproducibility.^6^

Following the successful implementation of WSI in Western countries, Indian researchers initiated its use in diverse patient populations. For instance, Rao et al. assessed concordance and time to diagnosis using WSI compared with light microscopy in the context of prostate carcinoma. They observed no major discordance and a high intra-observer agreement for both primary diagnosis (κ = 0.9) and Grade group (κ = 0.7–0.8).^37^ The study findings indicated that WSI can be as effective as traditional glass slide microscopy for the primary histological assessment/diagnosis of prostate biopsies. Gupta et al. graded tubular atrophy in 151 native kidney biopsies using conventional eyeballing and WSI-based digital measurement, treating the latter as the reference standard. The overall concordance between the two methods was 66.22%, with eyeballing over-grading tubular atrophy in 30.46% of cases, particularly in grades I and II, where sensitivity was only 52% and 47.83%, respectively. The authors concluded that eyeballing over-assesses tubular atrophy relative to accurate digital quantification, and that WSI represents a more reliable and reproducible method for this assessment in routine clinical practice.^38^ The authors noted that with further technological advancements, WSI could become a reliable method to assess tubular atrophy. In a study by Islam et al., 10 pathologists examined 30 diagnostically challenging cases of oral premalignancy and malignancy using both WSI and conventional techniques. WSI was associated with a significantly shorter mean time to diagnosis and with visualization of more fields in less time than traditional methods.^6^

Taken together, the Indian validation studies present a broadly consistent picture of WSI non-inferiority for primary diagnosis, though important nuances emerge on closer comparison. Studies conducted at tertiary oncology centers with dedicated IT infrastructure, trained histotechnologists, and high case volumes report near-perfect concordance, whereas studies conducted in less-controlled settings or involving more subjective grading tasks report lower agreement.^29^, ^37^, ^38^ This suggests that the performance of DP in the Indian context is strongly dependent on institutional readiness, including slide preparation quality, network reliability, and pathologist familiarity with digital platforms, factors that must be addressed systematically before broader implementation. With continued research and technological advancements, DP could meaningfully enhance diagnostic accuracy and accessibility, particularly in resource-limited settings.

#### AI-assisted diagnosis and personalized treatment

AI has transformed diagnostic workflows in DP by improving accuracy, efficiency, and reproducibility. Several AI applications in pathology have now been prospectively validated. These include automated tumor detection and segmentation in breast, prostate, and colorectal cancers; mitotic figure counting, which has demonstrated superior reproducibility compared to manual assessment; quantification of immunohistochemical biomarkers such as Ki67 proliferation index, PD-L1 expression, and HER2 scoring; and prediction of molecular features directly from hematoxylin and eosin (H&E)-stained slides.^39^, ^40^ Regulatory clearance of select AI tools by agencies such as the US Food and Drug Administration (FDA) and the European CE marking framework further underscores the clinical readiness of these applications, although equivalent regulatory pathways in India remain in early stages of development.^41^

WSIs, when paired with advanced computer vision algorithms, allow for feature extraction, object recognition, and predictive analytics. AI algorithms have been integrated into DP workflows as independent diagnostic systems, assistive tools, and automated quantifiers. This automation not only aids pathologists in diagnosis but also supports the prediction of disease progression, therapeutic response, and patient outcomes. Moreover, integrating histopathological data with clinical, genetic, and radiological information enables comprehensive “omics” analyses, overcoming the limitations of traditional evaluations.^39^, ^40^ Automated image analysis using algorithm-driven scoring systems also improves the accuracy of prognostic marker assessment.^42^ Therefore, AI-assisted diagnosis can benefit private labs, specialized research centers, and clinicians, aiding the development of personalized treatment plans. DP standardizes sample analysis, minimizing inter-observer variability inherent to conventional microscopy or grading methods. Rathi et al. found that uPath HER2 Dual ISH Image Analysis for identifying HER2 amplification was non-inferior to manual scoring and could support pathologists in routine diagnosis.^43^ In oncology, DP combined with AI and machine learning (ML) tools offers significant advantages in cancer risk assessment. AI helps quantify immunohistochemical biomarkers (e.g., Ki67 and PD-L1) and the detection of isolated tumor cells in lymph nodes with greater sensitivity and consistency.^39^, ^40^ Moreover, it standardizes subjective grading systems, such as the Gleason score (GS), for prostate cancer and breast cancer grading, reducing inter-observer variability. AI tools can classify patients into low-, intermediate-, or high-risk categories, guiding appropriate treatment strategies. Nagpal et al. observed that AI-based GS predictions were more effective at stratifying risk compared to pathologist-derived GS.^44^ Similarly, Shao et al. proposed an AI-driven prostate cancer risk stratification model, CCHEK (CAPRA-S 1 CNN H&E & Ki-67). Their findings highlighted CCHEK's superiority over manual assessment using CAPRA-S in accurately predicting patient outcomes.^45^

In neuro-oncology, AI models excel in glioma classification, tumor grading, and prognosis prediction, whereas semantic segmentation techniques link spatial tumor features to survival outcomes.^39^ Content-based image retrieval systems further empower pathologists to diagnose rare or complex conditions by enabling access to similar cases from extensive databases.^40^

Further, AI's predictive capabilities link histological features to genetic and molecular profiles, advancing the understanding of tumor biology and therapeutic response. ML-based convolutional neural networks play a pivotal role in identifying morphological patterns associated with prognostic outcomes, such as recurrence or metastasis risks.^39^, ^40^ Applications range from predicting *KRAS* mutations in colorectal cancer to assessing microsatellite instability.^40^ Whereas integrating genomic data with imaging is challenging due to the complexity of multidimensional datasets, AI advancements promise to unify morphological, molecular, and clinical information, paving the way for precision oncology. These applications can thereby minimize unnecessary molecular testing and improve cost-efficiency.^39^, ^40^

Beyond prognostic marker quantification, AI-based tools are increasingly being explored for their potential to identify predictive biomarkers directly from WSI, an application of particular clinical relevance in settings, where molecular pathology infrastructure is limited or absent. Computational pathology algorithms have demonstrated the ability to infer molecular features, such as microsatellite instability, directly from H&E-stained WSIs, without the need for additional immunohistochemical or genomic testing.^46^ In rural and resource-constrained labs in India, where access to fluorescence in situ hybridization, next-generation sequencing, or advanced molecular assays may be unavailable or unaffordable, such AI-driven biomarker prediction tools could serve as a critical first-line screening step, enabling a more targeted and cost-effective use of confirmatory molecular testing where it is truly needed.

AI-based triage systems represent another high-impact application in resource-limited settings. By automatically prioritizing cases based on predicted severity, malignancy likelihood, or diagnostic complexity, these tools can help pathologists, particularly those managing high case volumes with limited support staff, allocate their attention more efficiently. AI algorithms have been validated for the triage of cervical cytology and prostate biopsies, flagging high-risk cases for an expedited review.^47^, ^48^ In a country like India, where pathologist-to-population ratios remain critically low, intelligent case triage could meaningfully reduce diagnostic delays and improve patient outcomes.

Despite promising validation results, the integration of AI into routine pathology workflows requires careful consideration of deployment architecture. Most clinical implementations follow a “human-in-the-loop” model, wherein AI-generated outputs are reviewed and validated by pathologists before final diagnosis, ensuring clinical accountability and minimizing automation bias. Key challenges include model generalizability across diverse populations, variability in slide preparation, and data drift over time, which may degrade algorithm performance if not continuously monitored. In the Indian context, where heterogeneity in staining protocols, scanner types, and lab practices is substantial, the robustness of AI models becomes a critical determinant of clinical utility. Therefore, successful implementation requires continuous local validation, periodic recalibration using institution-specific datasets, and governance frameworks defining the role of AI in diagnostic decision-making.

### Storage and archival of data

Hospitals and medical centers generate a significant amount of digital medical images required for surgical planning and diagnostic assessments every day. These images necessitate large-scale storage and databases, particularly for maintaining registries, such as cancer registries or those focusing on rare or unique cases. Digital images are stored electronically, eliminating the limitations of the delicate glass slides used in conventional pathology.^3^ Digital slides can be viewed at any workstation, saving time on organizing and retrieving the slides.^26^ Further, it can free up space by eliminating the need for manual storage. The storage and archival of high-resolution data ensure efficient data management and retrieval, benefiting both private and public healthcare platforms.

In 2013, the Cancer Digital Slide Archive (CDSA) was established as an online platform to provide the digital images of patient specimens from The Cancer Genome Atlas (TCGA). This resource complements TCGA's genomic and clinical data by offering DP images of frozen and formalin-fixed paraffin-embedded (FFPE) biopsies.^49^ Frozen specimens were quality-checked for tumor purity and necrosis, whereas FFPE sections were archived for histological confirmation. Initially, the CDSA served as a comprehensive DP image repository, but data access required downloading large files. It was developed to improve accessibility by providing a more efficient storage and archival solution.^49^

Cancer imaging biobanks are emerging in India as collaborative efforts between the government and premier academic institutions, with the aim of creating a well-defined, annotated image database and fostering collaboration among AI researchers, clinicians, and industry.^50^

### Quality assurance

DP, with its transformative potential, necessitates robust quality assurance (QA) frameworks to maintain diagnostic accuracy and reliability. Integrated laboratory information management systems (LIMSs) further enhance QA through end-to-end traceability, real-time monitoring, and supporting adherence to clinical protocols.^51^ Some institutions from the United States have pioneered integrating DP into clinical operations, utilizing gap analyses and subsequent action plans to establish internal QMS protocols. Such systems ensure that clinical lab standards are upheld while adapting to the unique demands of digital workflows, such as histology quality checkpoints and system validations.^52^, ^53^ Tools like the point-of-use QA system also help address quality-related challenges by assessing the impact of environmental factors and device settings on image quality.^52^, ^53^ This indicates that DP supports compliance with rigorous regulatory standards through robust validation protocols.

In India, developing robust QA programs has multiple benefits. Widespread adoption of standardized programs may ensure consistent quality across labs, address variability in pathology services, and improve patient outcomes and equitable healthcare delivery nationwide. As its adoption accelerates, DP is poised to transform QA practices by integrating automation, remote collaboration, and data management, ensuring reliable, scalable, and high-quality pathology services.

### Diagnosis for frozen sections

A primary challenge in conventional FS diagnosis is the need for an on-site pathologist, especially in remote or smaller facilities. DP has significantly improved primary FS diagnosis by overcoming several limitations inherent to traditional pathology.^54^ For instance, a Canadian hospital reported diagnostic delays in the absence of a pathologist, impacting academic responsibilities and consultation opportunities, particularly for time-sensitive neurosurgical FS.^55^, ^56^ After switching to WSI, the time required to review an FS slide dropped significantly, from over 20 min with robotic microscopes to approximately 15 min per case. This faster TAT helped meet CAP accreditation benchmarks while maintaining diagnostic accuracy and offering superior image quality compared to robotic microscopes.^54^, ^55^, ^56^

The success of WSI for primary FS diagnosis is attributed to a variety of factors, including reliable, high-quality slides produced by skilled histotechnologists and the integration of a well-coordinated team involving pathologists, IT support, and surgeons. Despite occasional technical challenges, such as network issues or scanner malfunctions, WSI's overall performance has been effective. Careful planning, system validation, and dedicated personnel are crucial for successful WSI implementation, ensuring safe and efficient FS diagnosis and improved patient care.^54^, ^55^, ^56^

In 2023, expert oncologists from a leading cancer center in India conducted a prospective validation study of the diagnostic utility of WSI for remote reporting of FS using a portable digital scanner system and a consumer-grade computer.^57^ It was a first-of-its-kind study from a developing country like India. WSI showed a 97.6% (95%–99%) diagnostic accuracy and an almost perfect inter-observer (κ = 0.993) and intra-observer (κ = 0.987) agreement among four pathologists.^57^

In India, where the distribution of pathologists is often uneven, and many regions lack immediate access to specialized pathology services, the adoption of WSI for FS could help bridge the gap in underserved areas. It could also enhance healthcare delivery, particularly in critical areas such as oncology and surgery, where timely and accurate diagnoses are essential. Although multiple Indian studies report high diagnostic concordance and feasibility of DP, these findings are predominantly derived from tertiary referral centers with advanced infrastructure, standardized workflows, and trained personnel. In contrast, there is limited evidence from Tier 2 and Tier 3 labs, where variability in slide preparation, inconsistent staining quality, limited IT infrastructure, and lack of trained personnel may significantly impact diagnostic performance. This introduces a potential overestimation bias in current validation studies, which may not fully reflect real-world conditions across decentralized healthcare settings. Furthermore, whereas telepathology and remote reporting demonstrated feasibility during controlled environments such as the COVID-19 pandemic, long-term sustainability, cost-effectiveness, and scalability across heterogeneous healthcare tiers remain inadequately studied. These gaps underscore the need to move beyond feasibility-focused research toward implementation science frameworks that evaluate real-world deployment, system integration, and health outcomes across diverse Indian healthcare settings.

## Current scenario and impact of digital pathology in India

DP was first introduced in India in the 2000s and gradually accepted by some institutions, hospitals, and Indian diagnostic labs.^14^ A 2016 survey of 247 pathologists in India found that 98% reported a need for telepathology and DP, 34% used DP in routine practice, and 82% agreed that DP is useful for expert opinion.^58^

With the nationwide lockdown during the COVID-19 pandemic in India, remote working led to greater adoption of DP and telepathology.^23^, ^24^, ^25^ Tata Memorial Centre, Mumbai, validated remote WSI-based reporting and adopted reporting from home for cancer patients early during the pandemic in 2020.^23^, ^29^ Eighteen pathologists validated the use of DP for remote diagnosis in 567 biopsy cases. The Roche Ventana DP200 whole-slide scanner was used, and the findings revealed no major discordance and 1.2% minor discordance (*n* = 7/567). The absolute major concordance demonstrated the non-inferiority of WSI for remote diagnosis in a risk-mitigated environment. Despite a slightly longer evaluation time with WSI, the elimination of intermediate reporting steps with the DP workflow improved efficiency and reduced TAT.^23^

Other hospitals have also since employed remote reporting for glass slides and FSs, with some observing a 100% concordance between light microscopy and WSI.^24^, ^25^ Recently, two leading diagnostic lab chains in India announced the adoption of DP solutions across their branches to facilitate timely diagnosis and remote consultancy, indicating a trend to integrate AI to meet the growing demands of the healthcare system.^59^, ^60^ Apollo Hospitals launched a nationwide DP initiative in 2023 and SRL Diagnostics partnered with PathPresenter to enable DP consultations across its lab network. These institutional experiences provide proof of concept for broader implementation and offer lessons for scalability. Table 2 represents the strength of evidence for DP applications in India.

**Table 2:** Strength of evidence for digital pathology applications in India.

Domain	Evidence strength	Key insight
Primary diagnosis (WSI)	High	Consistent non-inferiority in tertiary centers
Telepathology	Moderate	Feasible but limited long-term scalability data
AI-assisted diagnostics	Moderate	Strong validation but limited real-world deployment
Cost-effectiveness	Low	Sparse India-specific economic evaluations
Rural implementation	Low	Significant evidence gap

AI: artificial intelligence; WSI: whole-slide imaging.

## Challenges and potential solutions for adopting digital pathology in India

The introduction and practice of DP and AI in India present a significant opportunity to enhance the healthcare sector and improve the standard of patient care. However, challenges like infrastructure and IT integration, financial constraints, and security concerns pose significant barriers to their successful adoption.^26^, ^61^ Overcoming these hurdles is crucial to facilitating the widespread application of DP in India. Addressing these barriers requires a phased, multistakeholder implementation framework organized across three horizons. In the near term (0–2 years), priority actions include establishing pilot programs at select tertiary centers to generate local validation data, developing standardized training modules for pathologists and IT staff, and mapping existing LIMS infrastructure to identify integration requirements. In the medium term (2–5 years), centralized scanning hubs serving clusters of peripheral labs offer a scalable and cost-effective model for extending DP access, supported by public–private partnership (PPP) financing arrangements and government digital health funding. In the long term (5+ years), sustainability depends on embedding DP literacy in residency training, building India-specific AI datasets, achieving regulatory clarity for AI-based diagnostic tools, and establishing national QA standards for DP services.

**Fig. 2:**
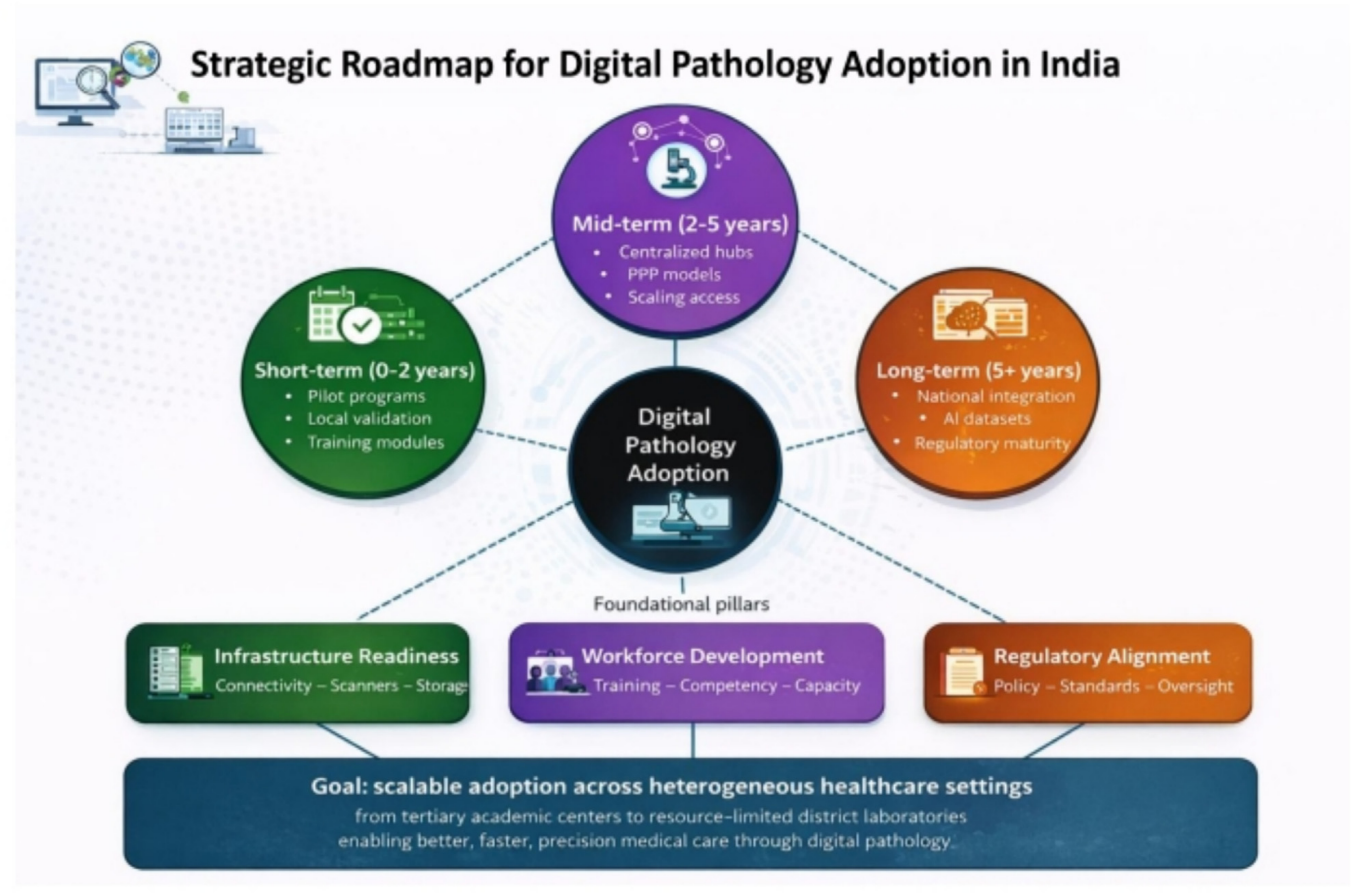
Three-tier implementation framework for digital pathology adoption in India. The framework outlines a phased approach across three time horizons: short-term (0–2 years), focusing on pilot programs, local validation, and workforce training; mid-term (2–5 years), emphasizing scale-up through centralized scanning hubs and public–private partnerships; and long-term (5+ years), targeting nationwide integration, development of India-specific AI datasets, and regulatory maturity. These phases are supported by three foundational pillars: infrastructure readiness, workforce development, and regulatory alignment, enabling scalable adoption across heterogeneous healthcare settings from tertiary centers to resource-limited labs.

To operationalize DP adoption in India, we propose a structured three-tier implementation framework integrating infrastructure readiness, workforce development, and regulatory alignment. This framework aligns short-term pilot deployment with long-term system-wide integration and is designed to be scalable across heterogeneous healthcare tiers, from tertiary academic centers to resource-limited district labs. A schematic representation of this framework is provided in Fig. 2. Whereas the following subsections and Table 3 address the key barriers and solutions across each of these dimensions.

**Table 3:** Barriers to digital pathology implementation in India.

Domain	Barrier	Potential solutions	Key stakeholders
Infrastructure & IT	Lack of physical space, WSI scanners, high-speed servers, and medical-grade monitors; connectivity gaps in Tier 2/3 and rural settings^26^, ^61^, ^67^	• Centralized scanning hubs serving clusters of peripheral labs^75^ • Adoption of DICOM standards and PACS-linked storage^69^ • JPEG2000 compression to reduce file sizes^68^ • Tiered storage: SSD for active cases, tape/cloud for archival^1^, ^63^ • Minimum 10 Mbps connectivity for remote sign-out; higher bandwidth for multi-user and frozen section workflows^29^	• Hospital/lab administrators • IT and health informatics professionals^64^ • Technology vendors • Government (NHM, ABDM)^79^, ^80^
LIMS integration	Legacy or heterogeneous LIMS lacking standardized interfaces; limited health informatics expertise^64^, ^66^	• Adopt HL7/FHIR and DICOM interoperability standards^66^ • Automate case assignment, specimen tracking, and audit trails via DP–LIMS integration^64^ • Train dedicated health informatics personnel^65^	• IT teams and LIMS vendors • Academic medical centers • Regulatory bodies (NABL)
Cost & financing	High capital costs: WSI scanner USD 100 K–400 K, displays USD 5.5 K–39.5 K, GPU servers USD 20 K–40 K, image analysis software USD 20 K+; recurring licensing and maintenance costs^58^, ^61^, ^70^, ^71^, ^72^	• PPP models: vendors provide infrastructure in exchange for data-sharing or revenue-sharing arrangements^75^, ^77^, ^78^ • Government subsidies via ABDM and National Health Mission^79^, ^80^ • Revenue diversification: remote consultations, digital biobanks, AI dataset commercialization • Shared service and subscription-based models for teaching institutes • Phased implementation to spread capital expenditure^32^, ^74^ • Breakeven achievable in 1–7 years in fully digital workflows^32^, ^76^	• Hospital management and finance • Technology vendors and diagnostic chains^75^ • Ministry of Health and NITI Aayog^79^, ^80^ • Research funding bodies (DST, ICMR)^70^
Workforce readiness	Resistance to DP adoption; unfamiliarity with digital navigation tools; reduced tactile feedback; fear of job displacement; ∼45% of physicians lack computational proficiency^3^, ^70^, ^81^, ^82^, ^84^	• Integrate WSI-based reporting and AI tool evaluation into pathology residency curricula (NBE/university frameworks) • Structured CME programs for practicing pathologists; IAPB and IAC to lead^83^, ^84^ • Hybrid workflows with structured validation period (parallel glass slide and digital reporting)^51^ • Peer mentorship from early adopters^83^, ^84^ • Task-shifting: trained technicians perform scanning under pathologist supervision^86^, ^87^ • Pathology informatics subspecialty development^88^ • AI framed as augmentative, not replacement, tool^83^, ^85^	• Pathologists and residents • IAPM and IAC (professional bodies) • Academic medical centers • National Board of Examinations • Technology vendors (training programs)
Data security & regulation	Patient data privacy risks; cybersecurity vulnerabilities (∼29 million patient records compromised in 2020); nascent SaMD regulatory pathway; no India-specific DP accreditation standard^72^, ^89^, ^92^	• Compliance with DPDP Act 2023 and DPDP Rules 2025 (data localization, consent, accountability) • Alignment with Telemedicine Practice Guidelines 2020^91^ • CDSCO guidance on software as a medical device (SaMD)^92^ • Decentralized storage and blockchain-based transaction validation^90^ • NABL accreditation: formal concordance studies and SOPs for WSI validation • Develop India-specific DP accreditation standards (analogous to CAP/RCPath) • ABDM as backbone for federated DP data exchange	• CDSCO and Ministry of Health^92^ • NABL and professional bodies • Hospital ethics committees^93^ • Legal and compliance teams • NITI Aayog^91^
Dataset & AI quality	Limited India-specific training datasets; AI models predominantly trained on non-Indian populations; risk of algorithmic bias and diagnostic errors from uncritical AI use^93^, ^94^, ^95^	• Multi-institutional data pooling: India Pathology Dataset,^96^ CHAVI cancer imaging biobank^50^, ^97^ • Prospective, randomized, multicenter trials in Indian patients^94^ • Fair representation learning to mitigate demographic bias^93^ • Independent AI ethics committees with pathology expertise^93^ • Pathology informatics divisions within institutions for AI output evaluation^88^	• Research institutes and academic hospitals^96^, ^97^ • AI developers and technology companies • Government (DST, DBT, ICMR)^94^ • Independent ethics committees • International collaborators^95^

ABDM: Ayushman Bharat Digital Mission; AI: artificial intelligence; CAP: College Of American Pathologists; CDSCO: Central Drugs Standard Control Organization; CHAVI: cancer imaging archive (chest imaging archive initiative context-specific); CME: continuing medical education; DBT: Department Of Biotechnology; DICOM: digital imaging and communications in medicine; DP: digital pathology; DPDP Act: Digital Personal Data Protection Act; DST: Department Of Science And Technology; FHIR: fast healthcare interoperability resources; GPU: graphics processing unit; HL7: health level seven; IAC: Indian Association Of Cytologists; IAPM: Indian Association Of Pathologists And Microbiologists; ICMR: Indian Council Of Medical Research; IT: information technology; LIMS: laboratory information management system; NABL: National Accreditation Board For Testing And Calibration Laboratories; NBE: National Board Of Examinations; NHM: National Health Mission; PACS: picture archiving and communication system; PPP: public–private partnership; RCPath: Royal College Of Pathologists; SaMD: software as a medical device; SOPs: standard operating procedures; SSD: solid-state drive; WSI: whole-slide imaging.

### Infrastructure and IT requirements

DP adoption in a healthcare facility or an institution requires a dedicated physical space for high-resolution WSI scanners integrated with AI and image management software, internal and external servers, and IT equipment.^32^ Further, high-resolution digital images require adequate storage capacity, ensuring servers can store images without compromising data quality.^62^ Compression or decompression of images during data transmission may lead to discrepancies in diagnoses among experts. It may also cause the loss of crucial data, hindering the development of accurate training sets for ML models.^63^ Consequently, expert IT assistance is necessary to maintain medical-grade monitors, implement robust security measures, deploy high-performance servers, and integrate DP with LIMS to streamline workflows.^64^

In a hybrid digital/analog workflow, where some cases are reported digitally, whereas others continue on glass, the informatics team plays a central coordinating role.^65^ This includes managing parallel case-tracking systems, ensuring that digital and glass-slide workflows do not diverge in TAT or documentation standards, and troubleshooting technical failures that could delay sign-out. Dedicated IT personnel are required to manage scanner uptime, monitor network performance, and maintain software integrations, responsibilities that extend well beyond conventional lab IT support.

Integration of DP systems with LIMS presents a particular challenge in the Indian context, where many labs operate on legacy or heterogeneous information systems that lack standardized interfaces. Seamless DP–LIMS integration is essential for automating case assignment, tracking specimen status, embedding digital images within pathology reports, and enabling end-to-end audit trails. However, the lack of inter-operability between proprietary DP platforms and existing LIMS, compounded by the limited availability of trained health informatics professionals, makes this integration technically and financially challenging for most Indian institutions. Adopting open standards such as Health Level Seven International (HL7), including Fast Healthcare Interoperability Resources (FHIR) and Digital Imaging and Communications in Medicine (DICOM) for pathology, can help bridge this gap, but implementing them requires significant institutional investment and expertise.^66^

Several public hospitals and health care centers, particularly in remote and suburban areas of India, lack the necessary infrastructure and IT support, with connectivity issues, such as a lack of network coverage, limited or unstable internet connectivity, and lower frequency coverage bands. These challenges are further exacerbated by low investment in technological upgrades due to decreased revenue.^67^ This may result in a longer TAT and misinterpretation in diagnosis, impeding workflow efficiency. Notably, Rao et al. reported issues with sign-out only when the network speed was <10 Mbps.^29^

To contextualize the scale of these infrastructure demands: a single uncompressed WSI file typically ranges from 1 to 4 gigabytes, depending on tissue size, magnification, and scanner resolution, with JPEG2000-compressed files averaging 300–800 megabytes.^1^, ^35^, ^68^ A lab processing 50 cases per day could generate upwards of 15–200 terabytes of image data annually, necessitating tiered storage architectures combining high-speed solid-state storage for active cases and lower-cost archival solutions for long-term retention. A reliable internet connectivity of at least 10 Mbps is required for uninterrupted remote sign-out, as demonstrated by Rao et al.,^29^ with higher bandwidths recommended for multiuser environments or real-time FS telepathology.

To address these challenges, sufficient cloud storage, competent image compression methods, and robust archival systems are essential.^63^ WSI images should conform to DICOM standards for smooth viewing and data transfer. However, WSIs often exceed the DICOM image object size limit. This has led to the development of softwares and protocols to link digital images with DICOM-based Picture Archiving and Communication Systems (PACS).^69^ Fast disk-based storage for immediate diagnostics and tape archives for long-term retention have proven effective. Compression methods, including lossy (JPEG2000) and lossless (TIFF), help reduce storage demands while preserving image quality. Additionally, adherence to relevant clinical guidelines, including the use of DICOM standards for image storage and exchange, ensures secure backup of digital slides and retrieval and manipulation without data loss. Labs often implement retention policies to manage storage efficiently, deleting short-term diagnostic slides after a set period while preserving educational and research slides indefinitely.^1^, ^22^

### Cost of DP implementation

According to a 2016 survey from India, approximately 68% of pathologists perceived the cost for DP implementation as a limitation.^58^ Installation of WSI systems, DP-related hardware and software, secure servers, and cloud storage necessitates a substantial investment from hospital or institutional funds.^61^ Initial costs include those for a single high-capacity (>100 slides) whole-slide scanner, ranging from 100,000 to 400,000 USD, and medical-grade displays, which cost USD 5500–39,500.^70^, ^71^ Software needs, based on the application, can further influence pricing.^70^ Image analysis softwares have a base cost of about USD 20,000, with additional features costing USD 100,000. A simple, non-redundant system with 3 months of online data storage for a lab producing 250,000 slides per year will cost around USD 90,000. Servers, especially graphic processing units to support high processing power for advanced softwares, algorithms, and AI-driven image analysis, cost up to USD 20,000–40,000.^70^ Moreover, recurring costs associated with cloud storage, software licensing and upgradation, hardware maintenance, and IT support contribute to the overall cost of DP implementation.^72^ Further, the smooth running of the equipment requires trained personnel and pathologists.^73^ Training these professionals to operate and maintain the equipment would add to the financial burden of budget-constrained health centers. These expenses could impede DP adoption in India, especially for small labs, hospitals, and diagnostic centers with limited funding.

The financial implications of DP adoption vary considerably depending on the institution's type and scale. For large private academic medical centers and high-volume diagnostic lab chains, which already operate established IT infrastructure and generate sufficient case volumes to justify investment, the business case for DP is relatively straightforward, with breakeven achievable through efficiency gains and revenue diversification.^32^, ^70^, ^74^ In contrast, small to mid-sized private diagnostic labs face a more challenging calculus, where the high capital cost of scanners and servers may be disproportionate to anticipated returns in the absence of scale. For public sector institutions, including government medical colleges, district hospitals, and primary care centers, the cost barrier is most acute, as capital budgets are constrained and fee-for-service revenue is limited. These institutions are most dependent on government subsidies, PPP arrangements, or shared-service models such as centralized scanning hubs to make DP economically viable.^75^ Academic institutions, regardless of public or private status, may additionally offset costs through research grants, AI development partnerships, and the monetization of teaching datasets, revenue streams less accessible to purely diagnostic labs.^70^

Cost-efficiency analyses for DP are limited and primarily based on single-center academic experiences, making generalization challenging. Studies suggest that cost-effectiveness is achievable only in fully digital workflows, with savings stemming from reduced glass slide handling, immunohistochemistry use, and labor costs.^76^ However, the breakeven point varies, influenced by productivity improvements and setup costs, with estimates ranging from 1 to 7 years.^32^ Further research on the cost-effectiveness of DP in the Indian setting is necessary.

Several revenue-generating strategies can be considered to justify the high investment associated with DP. A promising revenue opportunity for DP lies in remote pathology consultations to national and international clients. By offering expert second opinions, medical institutions can expand their market reach and monetize these services. Collaborative reporting and sharing of slides between labs can also generate revenue. Institutions can monetize digital copies by charging their use in patient consultations, teaching, and research purposes. Additionally, the sharing of anonymized data and forming partnerships with third-party algorithm developers can facilitate the creation of AI tools, providing a lucrative revenue stream through commercialization. Creating digital biobanks with subscription-based access for teaching institutes and offering external quality assessment programs for pathology labs can also generate additional income.

Beyond institutional revenue strategies, PPP models offer a viable pathway to subsidize the substantial upfront costs of DP adoption, particularly for government-run hospitals and health centers in rural and semi-urban India.^75^ Under such frameworks, private technology vendors or diagnostic chains could provide DP infrastructure, including scanners, softwares, and connectivity solutions, in exchange for data-sharing agreements, co-branding opportunities, or service-based revenue models. Precedents for such arrangements exist in other areas of India's digital health ecosystem, including telemedicine and radiology.^77^, ^78^ Additionally, government initiatives such as the Ayushman Bharat Digital Mission (ABDM) and the National Digital Health Blueprint provide a policy foundation for integrating DP into public healthcare delivery, and targeted funding through schemes like the National Health Mission could be leveraged to support pilot implementations at district and tertiary referral hospitals.^79^, ^80^

The savings in space and manpower management can translate into lower overhead costs, which, over time, contribute to greater financial efficiency. Faster TATs made possible by DP can also save costs for all stakeholders, including healthcare providers and patients, ultimately justifying the investment. By adopting these strategies, healthcare institutions can make a strong case for the return on investment in DP, helping to offset initial expenditures while contributing to ongoing financial sustainability.

### Workforce challenge

Despite having one of the highest numbers of pathologists globally, India faces a critical shortage of well-qualified MD pathologists to meet the demands of its rapidly expanding diagnostic pathology sector.^13^ Moreover, many pathologists continue to rely on traditional methods and resist adapting to modern pathology approaches, creating an adoption barrier that can hinder the transition to digital systems.^70^ Resistance to DP adoption among pathologists is multifactorial. Beyond generational or habitual preference for glass slide microscopy, specific barriers include unfamiliarity with digital navigation tools, such as panning, zooming, and focus adjustment on a screen, reduced tactile feedback compared to the microscope, initial discomfort with screen-based diagnosis in the absence of validated personal ergonomic setups, and uncertainty about the reliability of AI-generated recommendations.^81^, ^82^ Studies on healthcare professional adoption of digital tools suggest that hands-on training, peer mentorship from early adopters, and gradual exposure through hybrid workflows are more effective than didactic instruction alone in building confidence and competence.^83^, ^84^ Institutional policies that allow pathologists a structured validation period, during which parallel glass slide and digital reporting are conducted, can also help build trust in the digital platform before full transition.^51^

Clinicians may lack a sufficient understanding of DP's benefits, whereas limited education and awareness among pathologists can lead to concerns about job displacement as DP and AI are integrated.^64^, ^84^ However, emerging evidence supports the role of AI as an augmentative rather than replacement tool in pathology.^83^ Studies have demonstrated that AI-assisted workflows enhance diagnostic efficiency and reduce error rates, whereas the pathologist retains interpretive authority and clinical judgment.^85^ This paradigm shift, from job displacement to role transformation, reframes AI as a collaborator that expands the pathologist's capabilities, particularly in high-volume or resource-limited settings.

The adoption of DP in the Indian landscape requires an understanding of digital technologies and diagnostic decisions supported by AI-based softwares. Whereas AI support enhances diagnostic accuracy, there remains a risk of diagnostic errors if AI recommendations are followed without scrutiny.^3^ Globally, approximately 45% of physicians lack the computational proficiency necessary to transition to digital health technologies.^3^

Hence, there is an urgent need for structured and targeted training programs that enable pathologists to navigate the complexities of digital platforms and fully harness their potential. Pathologists must be trained, or the diagnostic team must find an ally, such as a computational expert, to critically evaluate AI-generated outputs, such that diagnostic decisions are both informed and independent of overreliance on AI. To address this, institutions must develop AI literacy in pathologists and create a division for Pathology Informatics to enable the evaluation of AI recommendations and provide accurate diagnoses.

A sustainable long-term solution to workforce readiness lies in integrating DP literacy into formal pathology training. Incorporating WSI-based reporting, AI tool evaluation, and digital workflow management into pathology residency curricula, aligned with the competency frameworks of the National Board of Examinations and university postgraduate programs, would ensure that future pathologists enter practice already proficient in digital platforms. Equally important is the development of structured continued medical education programs for practicing pathologists, enabling upskilling without disrupting existing clinical workflows. Professional bodies such as the Indian Association of Pathologists and Microbiologists and the Indian Academy of Cytologists are well-positioned to lead such initiatives in collaboration with academic medical centers and technology vendors.

Evidence-based models for DP workforce development are emerging. Task-shifting frameworks, in which trained lab technicians or pathology assistants perform slide preparation, scanning, and preliminary digital triage under pathologist supervision, have been explored to extend the effective reach of qualified pathologists in resource-limited settings.^86^ In some LMIC contexts, community health workers have been trained to operate portable scanning devices, with images transmitted to remote pathologists for interpretation, effectively decoupling the site of specimen collection from the site of diagnostic expertise.^87^ Role evolution is also evident at the specialist level: the emergence of the subspecialty of pathology informatics, encompassing expertise in AI tool validation, digital workflow design, and data governance, represents a new career pathway that Indian pathology training programs are only beginning to recognize.^88^ Encouraging early exposure to these roles through elective rotations, fellowship programs, and industry partnerships will be essential to building the informatics workforce that large-scale DP implementation will require.

### Security issues: Data privacy, cybersecurity, and legal compliance

The generation of high-resolution images of histological tissue sections represents the transfer of confidential patient data into a digital format. Digital data can be stored on-premises on servers owned by the institutional authority, in cloud-based servers managed by third-party service providers, or in a hybrid form. This raises concerns about data privacy and security, as well as the moral obligation to protect patient privacy. In 2020, the American Medical Association reported that the data of ∼29 million patients were compromised owing to unauthorized access, underscoring the significant data security challenge.^89^ Decentralized storage and transaction validation can help maintain data security.^90^

The General Data Protection Regulations in European countries offer a comprehensive framework, particularly in terms of upholding transparency and safeguarding individual rights.^72^ The Digital Personal Data Protection (DPDP) Act, 2023, is India's landmark law for protecting individuals' digital data, focusing on consent, transparency, and accountability for entities handling data (Data Fiduciaries). The DPDP Rules, 2025, were notified in late 2025, bringing key provisions into force. This step to safeguard patient data, address security concerns, and build practitioner confidence would encourage the adoption of DP.

In addition to data protection legislation, several Indian regulatory and policy frameworks are relevant to DP adoption. The Telemedicine Practice Guidelines, issued jointly by the Board of Governors of the Medical Council of India and NITI Aayog in 2020, provide the foundational framework for remote medical consultations, including remote pathology reporting.^91^ The Medical Devices Rules, 2017 (amended 2020), govern AI-based diagnostic software classified as medical devices, and the Central Drugs Standard Control Organisation has begun issuing guidance on software as a medical device, though a comprehensive, pathology-specific regulatory pathway remains to be defined.^92^ The ABDM further establishes a federated digital health infrastructure that could, in principle, accommodate DP data exchange across institutions. Alignment of DP implementations with these frameworks will be essential for regulatory compliance and for building institutional and patient confidence in digital diagnostic services.

From an accreditation standpoint, labs seeking National Accreditation Board for Testing and Calibration Laboratories certification, the primary quality accreditation for medical labs in India, are required to demonstrate the analytical validation of any new diagnostic method, including WSI-based reporting. This necessitates formal concordance studies comparing digital and glass slide diagnoses across a representative case mix, documented standard operating procedures for the digital workflow, and ongoing internal QA measures. As DP adoption grows, the development of India-specific accreditation guidelines for DP, analogous to those issued by the College of American Pathologists (CAP) or the Royal College of Pathologists, will be an important priority for national professional bodies and regulatory authorities.

Further, informed consent empowers patients to control their medical information and make informed decisions. Patients must be aware of the implications of their medical information being digitally analyzed or used in AI-driven diagnostics. Another ethical issue is the unfairness caused by bias in data, which arises from the lack of use of multiple ethnic groups' data required to develop AI algorithms.^93^ To address this, fair representation learning should be adopted to train algorithms on diverse datasets to mitigate biases. Moreover, independent ethics committees, with expertise in AI-based research, are crucial for overseeing AI development and ensuring transparency and accountability.

Despite the emergence of multiple regulatory and policy frameworks, significant ambiguity remains regarding the classification, validation, and approval pathways for AI-based diagnostic tools in India. Unlike established systems such as the FDA or CE marking frameworks, India currently lacks a clearly defined, pathology-specific regulatory pipeline for computational diagnostics. This uncertainty may delay clinical adoption, limit institutional investment, and create barriers for technology developers. Addressing these gaps through dedicated national guidelines and standardized validation protocols will be critical to enabling safe and scalable integration of DP and AI into routine clinical practice.

### Developing high-quality datasets

Algorithm-generated reports rely on the quality of datasets used to train the ML models. Creating large, high-quality clinical datasets is essential. AI-generated results in India face challenges due to limited training on local patient data and the unavailability of high-quality datasets. Advanced ML models and innovative algorithms can enhance the data accuracy and reliability of AI recommendations. Prospective, randomized, multicenter trials in Indian patients can improve the reliability of AI-based diagnostic and therapeutic recommendations in India.^94^

The lack of data specific to the Indian population can be addressed through collaboration among institutes. This approach enables data pooling to create large training datasets for robust models. For example, the Cancer Imaging Archive, an openly accessible repository, provides high-quality, well-curated imaging datasets to support cancer research.^95^ An Indian collaborative effort involving research centers, hospitals, and educational institutes has led to the creation of the India Pathology Dataset.^96^ Although still in its nascent stage, this DP platform paves the way for future development and awareness of DP and AI in India. Additionally, similar collaborative initiatives for cancer imaging biobanks are ongoing and could make research more relevant to patients in India.^50^, ^97^

Despite increasing evidence supporting the adoption of DP in India, several critical gaps continue to impede its widespread and equitable implementation. A key challenge is the absence of a clearly defined, pathology-specific regulatory and validation framework for an AI-based diagnostic tools. Current regulatory pathways remain fragmented and underdeveloped, lacking an India-specific equivalent to established systems such as the United States FDA or CE marking, thereby creating uncertainty in the classification, clinical validation, and approval of computational diagnostics. This ambiguity has the potential to delay clinical integration, limit institutional investment, and hinder innovation among technology developers. In addition, context-specific cost-effectiveness data are scarce, and the breakeven timeline for DP adoption, particularly in low-volume or resource-constrained settings, has not been adequately established. The development of large, demographically representative Indian pathology datasets-critical for training and external validation of AI models-remains in its nascent stages, raising concerns regarding generalizability and potential bias. Furthermore, optimal implementation models for DP in Tier 2 and Tier 3 settings, where infrastructural constraints are most pronounced, have yet to be defined through systematic pilot evaluations. Addressing these challenges through the development of standardized national guidelines, robust validation frameworks, targeted health economic analyses, and coordinated multi-institutional efforts will be essential to facilitate the safe, scalable, and contextually appropriate integration of DP and AI into routine pathology practice in India.

## Conclusion

The integration of DP and AI into the Indian healthcare ecosystem offers a transformative pathway to enhance diagnostic accuracy, reduce TATs, and expand equitable access to specialist services, particularly in underserved settings. Realizing this potential, however, requires a coordinated and structured response to persistent barriers, including infrastructure limitations, high capital investment, workforce readiness, and evolving regulatory ambiguity. To accelerate adoption, three priority actions emerge: (i) implementation of phased pilot programs with robust local validation across diverse healthcare tiers to generate context-specific evidence and de-risk scale-up; (ii) development of nationally aligned regulatory and accreditation frameworks specific to DP and AI-enabled diagnostics to ensure standardization, safety, and trust; and (iii) targeted investment in workforce training and digital infrastructure, particularly in Tier 2 and Tier 3 regions, to enable equitable and scalable deployment. Embedding these efforts within pathology training curricula and continuing medical education will further support technical proficiency and clinical acceptance of AI as an augmentative tool.

Achieving sustainable, system-wide integration will depend on aligning these priorities through coordinated PPPs and national digital health initiatives that support financing, infrastructure expansion, and implementation at scale. Concurrently, proactive engagement with evolving frameworks on data governance, telemedicine, and AI-based medical devices is essential, alongside the development of high-quality, annotated, India-representative datasets to ensure the validity and generalizability of AI models. Future research must prioritize real-world implementation studies and cost-effectiveness analyses to bridge the gap between feasibility and routine clinical adoption. Collectively, sustained multistakeholder collaboration will be critical to enabling a phased yet accelerated transition from early adoption to nationwide integration, ultimately strengthening the quality, efficiency, and equity of diagnostic services in India.

## Declaration of generative AI and AI-assisted technologies in the manuscript figure preparation process

During the preparation of this work, the author(s) used Images from ChatGPT in order to generate Fig. 1, Fig. 2 for illustration purposes. After using this tool, the author(s) reviewed and edited the content as needed and take(s) full responsibility for the content of the published article.

## Funding

This research did not receive any specific grant from funding agencies in the public, commercial, or not-for-profit sectors.

## Declaration of competing interest

The authors declare no competing interests relevant to this manuscript.
